# Byford on the Uterus

**Published:** 1871-08

**Authors:** 


*Byford on the Uterus.* A treatise on Chronic Inflammation and Displacements of the Unimpregnated Uterus. Second edition, enlarged with numerous Illustrations.

It is but a few months since we had the opportunity to notice the appearance of the first edition of this work, and feel gratified that the merit of the book should have been so well appreciated by the profession as to have a second edition so soon called for. There is no occasion for our going into any detailed account of the teachings of the work; it will be quite sufficient for our readers when we announce that the second edition has made its appearance. The chapters are upon such subjects as the following, viz:—" Symphatic accompaniments of Uterine diseases; Local Symptoms; Etiology; Prognosis; Complications of Inflammation of Cervix; Position of Inflammation; Progress and Termination; Diagnosis; General Treatment; Local Treatment; Nitrate of Silver and its substitutes; Treatment of sub-mucous Inflammation; Displacements, their Philosophy and Treatment.

These and kindred subjects are discussed in a masterly manner, without any show of superior knowledge or pretension as a discoverer. Dr. Byford writes the exact present state of medical knowledge on the subjects presented, and does this so clearly, so concisely, so truthfully and so completely, that his book on the uterus will always meet the approval of the profession, and be everywhere regarded as a popular standard work.

